# Choline Chloride-Based DES as Solvents/Catalysts/Chemical Donors in Pharmaceutical Synthesis

**DOI:** 10.3390/molecules26206286

**Published:** 2021-10-18

**Authors:** Rosa Amoroso, Frank Hollmann, Cristina Maccallini

**Affiliations:** 1 Department of Pharmacy, University of Chieti, Via dei Vestini 31, 66100 Chieti, Italy; cristina.maccallini@unich.it; 2 Department of Biotechnology, Delft University of Technology, van der Maasweg 9, 2629 HZ Delft, The Netherlands; f.hollmann@tudelft.nl

**Keywords:** NADES, choline chloride, pharmaceuticals, solvent, catalyst

## Abstract

DES are mixtures of two or more compounds, able to form liquids upon mixing, with lower freezing points when compared to the individual constituents (eutectic mixtures). This attitude is due to the specific hydrogen-bond interactions network between the components of the mixture. A notable characteristic of DES is the possibility to develop tailor-made mixtures by changing the components ratios or a limited water dilution, for special applications, making them attractive for pharmaceutical purposes. In this review, we focused our attention on application of ChCl-based DES in the synthesis of pharmaceutical compounds. In this context, these eutectic mixtures can be used as solvents, solvents/catalysts, or as chemical donors and we explored some representative examples in recent literature of such applications.

## 1. Introduction

Within the framework of green chemistry, also known as sustainable chemistry, solvents play a very strategic role, considering their importance as reaction media and in purification processes. Classical organic solvents often have a large number of intrinsic drawbacks; in fact, they are generally volatile, flammable, and hardly degradable. For human beings, long-term exposure can lead to chronic toxicity, and deleterious effects on respiratory, hematological, and thyroid functioning [1].

In the last decades, a large number of studies have been conducted to replace classical organic solvents with new sustainable ones, with the aim to improve the protection of environmental and human health [2]. This approach comprises the use of different systems, such as the deep eutectic solvents (DES), first described by Abbott et al. as potential alternative solvents to ionic liquids (ILs) [3]. When a DES is composed by natural origin components, it is further defined as a natural deep eutectic solvent (NADES) [4]. DES/NADES are selected mixtures of two or more components, namely hydrogen-bond acceptors (HBA) and hydrogen-bond donors (HBD), which have the capability to form eutectic mixtures with non-ideal behavior, characterized by a greatly depressed melting point relative to their components. The solid–liquid equilibria and eutectic temperatures of DES are the result of a combination of melting points, enthalpies of fusion, and intermolecular attractions. In particular, the negative deviation to thermodynamic ideality arises from the formation of strong bond interactions between the DES precursors [5].

Additionally, the peculiar properties of DES with respect to volatile solvents are the result of an intense network of intermolecular forces, mostly hydrogen bonds between the constituents, that makes these structures unique. Several authors have tried to rationalize the melting point depression, underlining the key role of the hydrogen bond on the system entropy; in DES, the complexation of the HBA anion, through an ionic hydrogen bond by the HBD, generates a delocalization of the negative charge, an increase in the size, and a weaker interaction with the cationic counterpart in the HBA. Thus, the involvement of a HBD delocalizes the molecular lattice of HBA, causing a melting point depression, with a direct relationship between the freezing temperature and the network of hydrogen bonds [6,7]. Typical DES physicochemical parameters, such as high viscosity and density, can be rationalized by the “hole theory”, proposed by Abbot et al. [8]. This phenomenological model assumes that, on melting, an ionic material contains empty spaces (holes) with random sizes and locations, continuously arising by variations in density. In DES, the complexation of the anions results in an increase of size; since the holes are smaller at low temperatures, large-sized ions move with difficulty in these systems. Thus, the involvement of a HBD delocalizes the molecular lattice of HBA, causing, for example, an increase of viscosity.

DES can be divided into various classes, based on the nature of the HBA and HBD used in their preparation: quaternary ammonium salts and anhydrous metal halides (type I), quaternary ammonium salts and hydrated metal halides (type II), ammonium quaternary salts and neutral organic compounds (type III), metal chloride salts and neutral organic compounds (type IV), and mixtures non-ionic species (type V) [9,10]. Within these four DES classes, single components can be combined to form binary or ternary eutectic mixtures. 

NADES are a specific subgroup of DES composed of naturally occurring components, such as sugars, organic acids, alcohols, amino acids, urea, choline chloride, and water. This denomination, coined by Choi et al., is not used by all scientists; thus, it is possible to find eutectic mixtures described as DES which fit in the definition of NADES. In biological systems, the NADES could play a role as a third type of medium, an alternative to water and lipids, involved in the biosynthesis, storage, and transport of poorly water-soluble biomolecules, but also in the survival of organisms at prohibitive low-temperature conditions [11]. NADES are particularly attractive because they are characterized by low volatility, a liquid state even in sub-zero temperatures, biodegradability, solute stabilization, stability to air, and easy preparation. Then, it is not surprising that they have found interesting applications in various areas of chemistry, such as in electrochemistry, synthesis, biocatalysis, analytical chemistry, and innovative technologies of extraction [12,13,14,15]. One of the main advantages of DES is that their properties can be modulated by changing the component ratios or by water dilution, developing tailor-made mixtures for appropriate applications; in this way, a decrease in viscosity can also be achieved [16]. 

Many studies consider that DES are nontoxic, based on the fact that most of the components forming DES are natural products. However, the toxicity of the final mixture must be carefully assessed since significant differences from the individual constituents might be observed [17]. Toxicity and biodegradability evaluations can be carried out by different approaches, and this could affect the obtained results. For example, it was reported that the acetylcholine chloride (AcChCl)/acetamide (1:2) DES toxicity can be due to acidification of the cell’s media, due to DES hydrolysis. However, this cytotoxic effect occurred only at high concentrations, higher than 600 mM [18]. Therefore, if on one hand the ecotoxicological profile of DES is not well defined and further evaluations are necessary, on the other they appear as a safer solvent with respect to conventional organic ones [19].

The low toxicity associated with the single components of DES makes these solvents very appropriate for pharmaceutical applications. One of the first applications in this field was the use of DES to increase drug solubility, with the aim to improve the pharmacokinetic aspects like absorption and excretion. This concept was developed up to the definition of the so-called therapeutic deep eutectic solvents (THEDES), in which one component of the eutectic mixture is a drug [20]. Still remaining in the pharmaceutical field, another important aspect of sustainability is the use of appropriate solvents during drug synthesis. Concerning this, some pharmaceutical companies have recently expanded their solvent selection guides with the aim to reduce the use of the most hazardous solvents [21]. DES fit well in this context, in which the researchers try to optimize the conditions of organic synthesis from a green point of view. In particular, the properties of high thermal stability, low vapor pressure, and the possibility of acting both as catalysts and as reaction media make these solvents promising candidates for innovative drug syntheses. Thanks to the possibility of tuning their chemical and physical properties, the DES also show high versatility in the efficient creation of complex pharmaceutical scaffolds. One last very important aspect is the low monetary impact of DES, which is an important feature thinking of production on an industrial scale.

A great deal of published methodologies involve one-pot or multicomponent reactions, with a significantly reduced number of solvents needed for intermediate purification [22]. In this context, we focused our attention on published studies in which choline chloride (ChCl)-based DES are used as solvents, solvents/catalysts, or chemical donors in the synthesis of pharmaceutical compounds. Here we present a literature summary covering representative examples.

## 2. Structure and Properties of Choline Chloride-Based DES

The most studied DES contain ChCl (2-hydroxyethyl-trimethylammonium chloride), which is a naturally occurring bifunctional compound containing both a quaternary ammonium salt and alcohol. It is an essential nutrient, commonly grouped with the B-complex vitamins, that plays key roles in methyl group metabolism, carcinogenesis, and lipid transport [23]. In eutectic mixtures, ChCl functions as HBA with a variety of HBD, such as urea, alcohols, sugars, hydroxyacids, and aminoacids, due to the simple preparation, nontoxic nature, good biodegradability, and preservation of bioactivities of target compounds [24]. Most interestingly, ChCl-based DES offer the advantage to be modulated in viscosity, pH, and polarity for their applications in health-related areas, such as pharmaceuticals, foods, and cosmetics [25]. The main limitations of these solvents are the significantly high viscosities and densities in comparison with water and common organic solvents, which can reduce mass and energy transfer during chemical reactions; water or glycerol addition and mild heating contribute to the disruption the hydrogen-bond network and weaken the intermolecular forces, although keeping the solvent supramolecular structure [26].

The structure of ChCl-based DES have been explored by several experimental techniques such as nuclear magnetic resonance (NMR), crystallography, mass spectrometry (MS), and infrared spectroscopy (FT-IR) [27]. Recently, computational studies have been conducted, allowing further knowledge of the supramolecular structure of these eutectic mixtures, showing that the hydrogen bonds or even the Van der Waals interactions interfere with the capacity of the initial compounds to crystallize [28]. In particular, density functional theory (DFT) demonstrated that, in ChCl-based DES, the electronegative anion chloride is capable to provide more binding sites to form hydrogen bonds, and a competitive activity was observed between various HBD and choline cation [29]. In this scenario, the formation of hydrogen bonds and the consequent delocalization of charges, in addition to designing the three-dimensional network, significantly decreases the melting points of the different eutectic mixtures.

ChCl-based eutectic mixtures could be classified into groups, based on HBD type: alcohol- and sugar-based, acid-based, amide-based, water-based, and ternary mixtures. Most of these mixtures are liquid at room temperature and can thus be used as solvents in many applications.

In alcohol- and sugar-based eutectics, the HBD are glycols, glycerol, and sugars. These solvents exhibit a neutral pH in the mixture, and this may be very useful for expanding applications [30]. A detailed hydrogen bond analysis performed by Perkins et al. on ChCl:ethylen glycol (*ethaline*) and ChCl:glycerol (*glyceline*) suggested that the predominant intermolecular hydrogen bond is formed by anion chloride-hydroxyl group of HBD, while the cation choline interactions seem to represent the smallest contribution to the extensive network of intermolecular forces [31]. Recently, Biernacki et al. reported an experimental and theoretical study of ChCl-based NADES with sorbitol and xylitol. In these solvents, the oversaturation of the HBD groups results in more extensive self-interaction compared to NADESs with ethylene glycol and glycerol, with higher viscosities and densities [32].

The acid-based mixtures consist of natural carboxylic acids, with characteristics similar to ionic liquids. Obviously, the carboxylic functions are hydrogen donors, and the freezing points are lower than 50 °C. One of the advantages of this type of solvent is the possibility to use them as acid catalysts; furthermore, varying the pH of the eutectic liquid, it is possible to design a solvent with specific properties and different catalytic activity [33]. Regarding the supramolecular structure, an instructive example is that of “*maline*” (ChCl:malonic acid), a NADES that exhibits an extensive amount of anion chloride–HBD and anion chloride–choline interactions, which drastically increase the bonding network and decrease the mobility of molecules within the mixture [31].

Among the amide-based, the most studied DES is a 1:2 mixture of ChCl and urea, named “*reline*”, firstly proposed by Abbott et al. in 2003 [3]. The melting point of the dry eutectic is 12 °C, substantially lower than the constituent pure components (ChCl mp = 302 °C, urea mp = 133 °C). The properties of *reline* have been extensively studied and rationalized, providing an explanation for the optimal ratio of 1:2 between ChCl and urea [34]. In a mixture of ChCl and urea, there is the possibility of an “*alphabet soup*” of hydrogen bonds, (ChCl ion pairs, urea, and chloride, choline, and urea, and urea–urea), in contrast with traditional molecular solvents which have a homogeneous hydrogen bond network. This peculiarity could be expected to increase the entropy of the system, with the decrease of the melting point [35]. The supramolecular structure of ChCl:urea 1:2 was described as a radially layered “*sandwich*”, where choline and urea work synergistically to bond with the chloride anion, allowing for the best distribution of charge between each component in a stoichiometrically determined fashion [36].

*Aquoline* is a eutectic solvent solely composed of ChCl and water in a 1:3.33 molar ratio, with a much lower viscosity than other common NADES. This mixture is prepared by mixing water and ChCl hydrate, and it is presently attracting considerable attention in electrochemistry [37]. A recent study by Triolo et al. identified for this “*uncoventional DES*” a complex structural scenario based on coordination of cation choline with water and anion chloride, dispersed in the bulk phase in which long chains of water are present, generated by interactions between water–water and water–chloride [38].

The addition of a third HBD to a DES causes the formation of a ternary mixture. Usually, the third component is water, and, although a physiological amount of water strengthens the hydrogen-bond network within the DES, amounts higher than 10% can weaken the interactions between each component of the mixture, generating variations of properties such as density and viscosity. In addition to water, which is the most frequent additive, glycerol, methanol, ethanol, and 2-propanol were also used [39]. The effects of these components on the supramolecular structure of DES were studied and furnished information especially for dedicated applications, where the structural features play a crucial role [40].

## 3. Choline-Based DES as Solvents/Catalysts in Pharmaceutical Synthesis 

### 3.1. Choline-Based DES as Solvents

As reported above, in the last years, DES have been utilized in organic synthesis as green media thanks to the high potential to replace the classical solvents. In fact, since the DES are characterized by a network of hydrogen bonds, they have the possibility to dissolve solutes that can form hydrogen bonds and stabilize transition states. Furthermore, during the purification procedures, the addition of water to a DES (very soluble in water) causes the precipitation of organic products, facilitating the workup and avoiding the use of solvents for the extraction. From the point of view of ecological footprint, most DES come from renewable sources and, thanks to their low vapor pressure, biodegradability, and almost non-existent toxicity, they are considered green solvents. In this section, some representative examples of building block synthesis in ChCl-based DES will be examined, with applications in medicinal chemistry. 

**Scheme 1:**
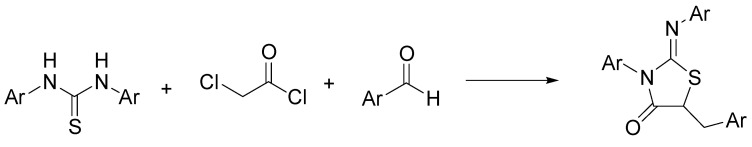
Condition reactions: ChCl/urea (12), 30 min, 80 °C (Ar = aryl).

The thiazolidin-4-one system is a privileged heterocycle present in many synthetic analogs with a wide range of pharmacological activities [41]. In particular, the 2-iminothiazolidin-4-one pharmacofore has been reported to exhibit anticancer properties that can be associated with the affinity for some biotargets [42]. Among the several synthetic routes available for the synthesis of the multifunctionalized 2-iminothiazolidin-4-ones, Mobinikhaledi and Amiri modified the classical pathway based on condensation between thioureas with chloroacetyl chloride, by introducing a one-pot three-component reaction in NADES, obtaining a variety of 5-arylidene-2-imino-4-thiazolidinones [43]. As shown in Scheme 1, they investigated the reaction of thioureas, chloroacetyl chloride, and aromatic aldehydes in ChCl/urea. The best results were obtained with condensation of *N*,*N*’-diphenylthiourea, chloroacetyl chloride, and benzaldehyde to give 5-benzylidene-3-phenyl-2(phenylimino) thiazolidin-4-one in 94% yield.

**Scheme 2:**
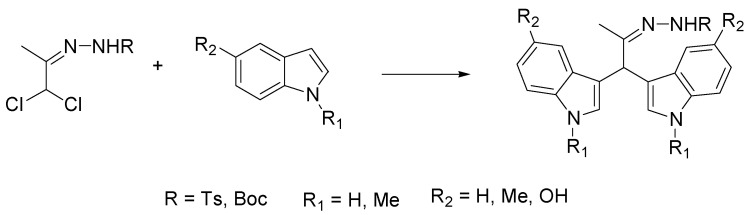
Condition reactions: H_2_O/[ChCl/glycerol (2:1)] (3:1), Na_2_CO_3_, 10–60 min, r.t.

The bis(indolyl)methanes (BIM) are indole derivatives with a broad spectrum of biological and pharmacological activities, present in various natural products and possessing important biological activity [44]. In particular, some BIM from cruciferous plants inhibit breast cancer cell proliferation by activation of multiple pathways, suggesting a potential role in the clinical treatment of ER-negative breast cancer [45]. The methods of BIM synthesis involve both one step as well as multistep strategies, based on the condensation of indoles and carbonyl compounds, using protic acids and Lewis acids as catalysts [46]. Recently, Grosso et al. reported the use of ChCl-based DES as green solvents to carry out a bis-hetero-Diels–Alder addition of nitroso- and azoalkenes with indoles [47]. The obtained BIM furnished, after hydrolysis, the corresponding carbonyl-BIM. Among the numerous reactions proposed, the best yields (50–80%) were obtained in the synthesis of hydrazonomethyl-BIM in H_2_O/[ChCl/glycerol (2:1)] (3:1) starting from alkylhydrazones (Scheme 2). 

**Scheme 3:**
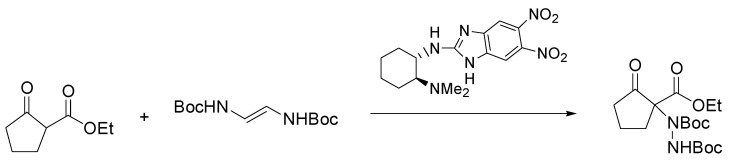
Condition reactions: ChCl/glycerol (1:2), 1 h, 25 °C.

Chiral 1,3-dicarbonyl compounds bearing an α-amine moiety represent an important structural motif present in natural compounds, most of them having biological and pharmaceutical activity [48]. Among the methods to gain access to this motif, significant developments have been reached in the asymmetric organocatalyzed amination of prochiral carbonyl compounds employing diazocarboxylates as nitrogen source [49]. Nigues et al. have improved the sustainability of this reaction by using ChCl/glycerol and ChCl/urea as the solvents and 2-aminobenzimidazole derivatives as catalysts [50]. As depicted in Scheme 3, the reaction of ethyl 2-oxocyclopentane-1-carboxylate with di-*t*-butyl-azodicarboxylate (DBAB) with a chiral 2-aminobenzimidazole derivative as catalyst under the optimized conditions gave the product of amination in excellent yield (94%) and good enantioselectivity (73% e.e.).

**Scheme 4:**
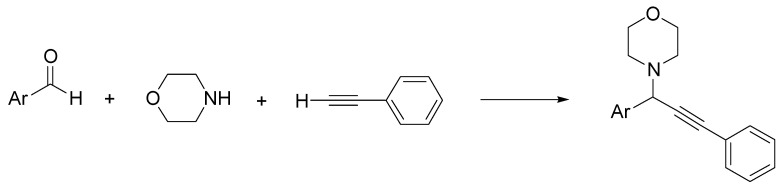
Condition reactions: ChCl/urea (1:2), CuCl (5 mol%), 15 h, 60 °C (Ar = aryl).

Propargylamines are alkyne coupled amine compounds used as skeletal structures in heterocyclic chemistry and drug design [51]. For example, rasagiline is a monoamine oxidase (MAO)-B inhibitor with neuroprotective and antiapoptotic effects, used in Parkinson’s disease and other neurodegenerative conditions [52]. Because of its versatility and wide use in organic synthesis, a number of methodologies have been developed for propargylamine derivatives, in particular three-component reactions of aldehydes, amines, and alkynes [53]. This method has been recently improved by substituting the classical solvents with environmentally friendly ChCl/urea [54]. First, the reaction between benzaldehyde, morpholine, and phenylacetylene in the presence of CuCl (5 mol%) as a catalyst was selected as a model to set the best conditions (solvent, catalyst amount, temperature, and time). Then, various aldehydes were used, and the best results were obtained with aromatic aldehydes with electron donor groups (38–90%) (Scheme 4).

**Scheme 5:**
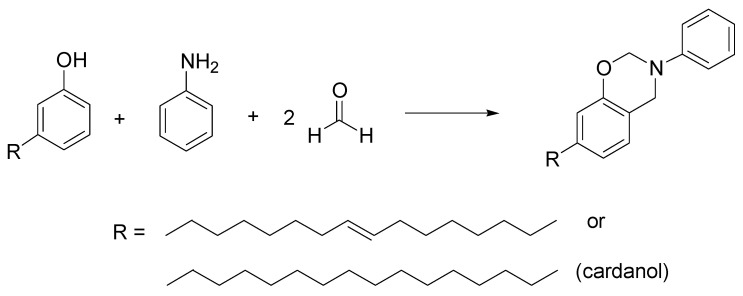
Condition reactions: ChCl/urea (1:2), 4 h, 70 °C.

Benzoxazines are compounds where an oxazine ring is condensed to a benzene ring, used in polymers, resins, and in the construction of interesting drug candidates as antilipidemics [55]. In this field, interesting compounds are the cardanol-derived benzoxazines. Cardanol is a natural oil with a phenolic structure, extracted from cashew or from Gingko biloba, extensively studied for its possible applications in industry and in pharmaceutical chemistry [56]. Behalo et al. developed a green Mannich-like condensation of a cardanol with its unsaturated derivative, aniline, and formaldehyde, in ChCl/urea as DES with good yields (81–88%) (Scheme 5). They also demonstrated the use of cardanol-based benzoxazines as scaffolds for the preparation of vesicular nanosystems [57]. 

**Scheme 6:**
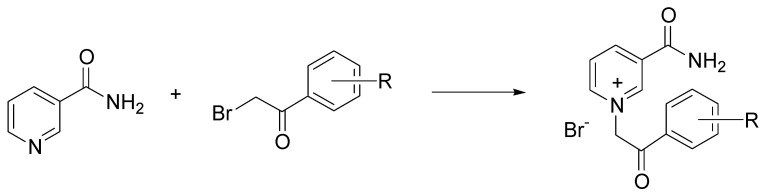
Condition reactions: ChCl/urea, ossalic acid, or levulinic acid, MW, 20 min, 80 °C.

Nicotinamide is an important naturally occurring heterocycle, which belongs to group B vitamins. Quaternary derivatives of nicotinamide have attracted much attention because of their antimicrobial, fungicidal, and cytotoxic properties [58]. The most common strategy for quaternization of nicotinamide is the nucleophilic substitution between pyridine and substituted 2-bromoacetophenones, called the Menschutkin reaction [59]. Busic et al. investigated the use of three ChCl-based DES in the quaternization of nicotinamide with 2-bromoacetophenones, performed by a conventional approach, microwaves (MW), and ultrasonic irradiation (blue). The highest yields were obtained by MW, and independently from the adopted solvent (Scheme 6) [60].

### 3.2. Choline-Based DES as Solvents/Catalysts

**Figure 1:**
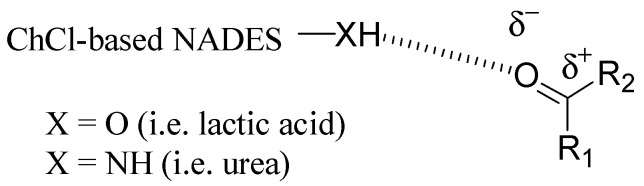
Activation of a carbonyl group by a ChCl-based DES/NADES as catalyst.

As described above, DES had great potential as a green alternative to classical organic solvents; in addition, in the last years, they have emerged as catalysts for various organic reactions, because they can be applied both as reaction media and as catalytic active species, providing homogeneous solutions. Although there are numerous examples of the catalytic effect of DES, this role has not been fully defined yet. To date, some mechanisms of catalysis have been proposed, mostly based on the formation of hydrogen bonds between the reactants and DES, thus facilitating the breaking of the chemical bonds involved in the reaction [33]. In this section, some representative examples of reactions carried out in ChCl-based DES working both as solvents and catalysts will be described. In reacting substrates, the function involved is always a carbonyl group, which is activated by DES thanks to its ability to establish strong hydrogen bonds, thus facilitating the subsequent electrophilic attack in reactions such as Michael additions, transesterifications, and condensations (Figure 1).

**Scheme 7:**
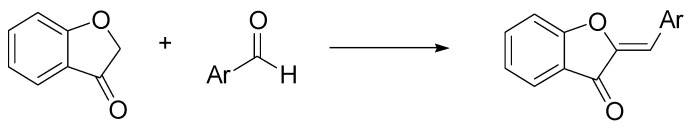
Condition reactions: ChCl/urea (1:2), 12-48 h, 80 °C (Ar = aryl).

Aurones, (*Z*)-2-benzylidenebenzofuran-3(2H)-ones, are a subclass of flavonoids that provide the golden-yellow color in many ornamental flowers. They exhibit a broad spectrum of activities including anticancer, antioxidant, antiparasitic, and antibacterial properties [61]. The simplest chemical synthesis of aurones is based on the condensation of a coumaranone with an aldehyde, under acidic, or basic conditions. Hawkins and Handy discovered a neutral condition for this condensation, based on the use of ChCl/urea, although with not excellent yields (max 73%) [62]. The reaction proceeds simply and cleanly by mixing the reactants in DES, and the desired aurones were recovered by extractive workup (Scheme 7). 

**Scheme 8:**
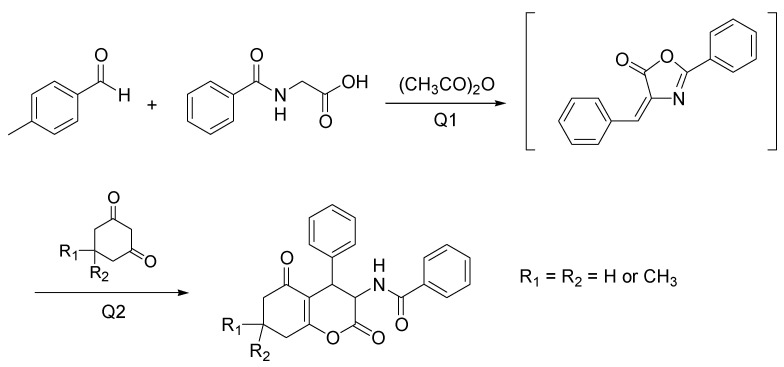
Optimized condition reactions: ChCl/urea (1:2), 120 °C, 40 min (Q1) and 10 min (Q2).

Novobiocin is a naturally occurring compound containing the aminocoumarin scaffold, with hopeful anticancer activity. Besides, other dihydrocoumarin and aminocoumarin derivatives possess significant bioactivities such as cytotoxic, anti-inflammatory, and antibiotic [63]. Despite the role as therapeutic agents, the available synthetic methods require toxic solvents or metal catalysts, with adverse effects on the environment. A new tandem approach to prepare the 3-aminohexahydroxcoumarins directly from aryl aldehydes, ippuric acid, acetic anhydride, and 1,3-dicarbonyl compounds, with ChCl/urea as a biorenewable solvent was developed by Zamani and Khosropour [64]. After preliminary studies in the bath, the reactions were conducted in a continuous micro-flow reactor, including two tubing glass (Q1 for aryl aldehydes, hippuric acid, and acetic anhydride, and Q2 for 1,3-dicarbonyl compounds) giving faster and quicker results. In Q1 an azalactone intermediate is formed, and then in Q2 the Michael addition of 1,3-dicarbonyl compounds gives rise to the target products (Scheme 8). 

**Scheme 9:**
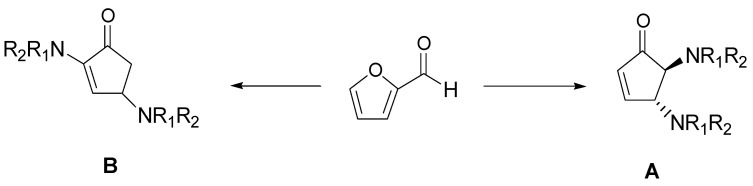
Optimized reaction conditions: ChCl/urea (1:2), 60 °C, 5 min (**A**) and 4 h (**B**).

The cyclopentenone moiety is a fundamental structural motif present in molecules such as prostaglandins, clavulones, and cephalotaxine esters, with significant anticancer activity [65]. Most of the reported synthetic strategies for the preparation of cyclopentenone require expensive catalysts, toxic solvents, and proceed with low yields. Therefore, there is an ongoing interest to develop new methodologies; in particular, erbium (III) chloride has found a good catalyst acting in green solvents, such as ethyl lactate [66]. Recently, Di Gioia et al. developed a simple and rapid method for the conversion of furfural into bifunctionalized cyclopentenones under green conditions by using the ChCl:urea, without any product purification [67]. They observed that reaction of furfural with secondary amines furnished, after 5 min, the bifuntionalized cyclopentenone **A**, that was totally converted to the structural isomer **B** after 4 h. These two conditions were applied for the synthesis of a series of derivatives, as shown in Scheme 9.

**Scheme 10:**
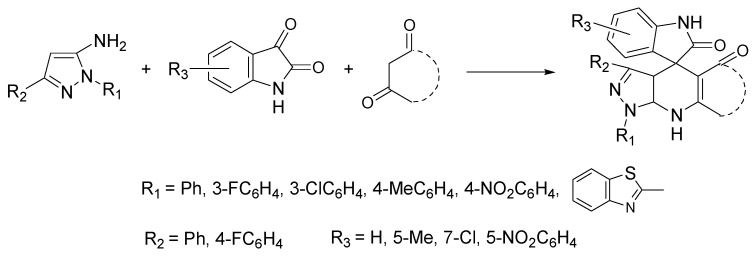
Reaction conditions: ChCl/lactic acid (1:2), MW, 60 °C, 15–30 min.

The merger of pyrazolo[3,4-b]pyridine and spirooxindole backbones into a hybrid structure (pyrazolo[3,4-b]pyridine spirooxindole) may result in interesting compounds with potential medicinal applications, since both scaffolds have remarkable pharmacological properties [68]. Of note is the anti-breast-cancer activity of some of these hybrid derivatives [69]. Zangh et al. proposed a sustainable protocol for the synthesis of pyrazolo[3,4-b]pyridines spirooxindoles with a variety of substituents through a one-pot three-component assembly between 1,3-aromatic-1H-pyrazol-5-amines, substituted isatins, and enolizable 1,3 dicarbonyl compounds. Excellent yields (82–95%) were obtained under microwave (MW) irradiation in ChCl:lactic acid as NADES (Scheme 10) [70].

**Scheme 11:**
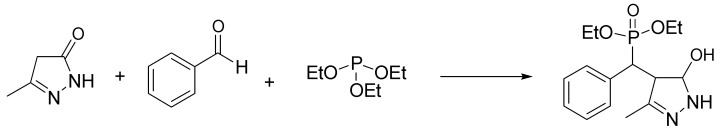
Condition reactions: ChCl/urea (1:2), 25 °C, 30 min.

Pyrazolylphosphonates are biologically interesting compounds containing phosphonate and pyrazole. They have received considerable attention due to their remarkable bioactivity profiles, with promising antioxidant and anticancer activity [71]. This interest has prompted the development of various synthetic approaches, including one-pot multicomponent reactions. Of interest is the condensation of pyrazolones with aldehydes and trialkyl phosphites, based on the in situ formation of benzylidene pyrazolone and subsequent reaction with a phosphorus nucleophile. Kalla and Kim have used as a catalyst for this one-pot synthesis a ChCl-urea mixture, with a series of advantages such as bio-friendly profile, mild conditions at r.t., and the avoidance of chromatographic purification [72]. The authors first developed a model reaction between 5-methylpyrazolone, benzaldehyde, and triethyl phosphite in mild conditions (Scheme 11). Then, the scope reaction was investigated using various aromatic aldehydes, pyrazolones, and trialkyl phosphites, allowing us to assess a clear improvement in product yields (90–96%) when using DES with respect to the other catalysts previously reported.

**Scheme 12:**
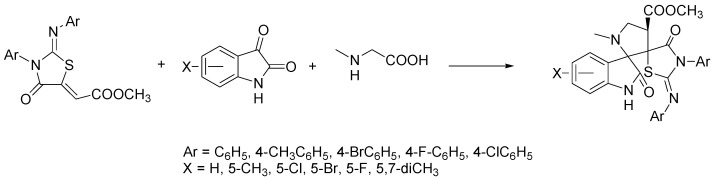
Condition reactions: ChCl/urea (1:2), 40–100 °C, 2–5 h.

From a synthetic chemistry point of view, the above illustrated 2-imino-4-thiazolidinones are very interesting scaffolds, due to the presence of electrophilic centers and activated exocyclic double bonds, and therefore they can behave like dipolarophiles in 1,3-dipolar cycloaddition reactions. In this context, Singh et al. have used 2-iminothiazolidin-4-ones as a core system for the construction of hybrids with spiro-oxindole-pyrrolidines as novel druglike molecules [73]. The preparation of hybrids was carried out with excellent yields (50–92%) and high regio- and stereo-selectivity by means of the multicomponent 1,3-dipolar cycloaddition of (2Z)-methyl-2-[(Z)-4-oxo-3-aryl-2-(arylimino) thiazolidin-5-ylidene] acetate as dipolarophile with substituted isatins and sarcosine, using ChCl/urea as the green solvent (Scheme 12).

### 3.3. Choline-Based DES as Chemical Donors

**Scheme 13:**
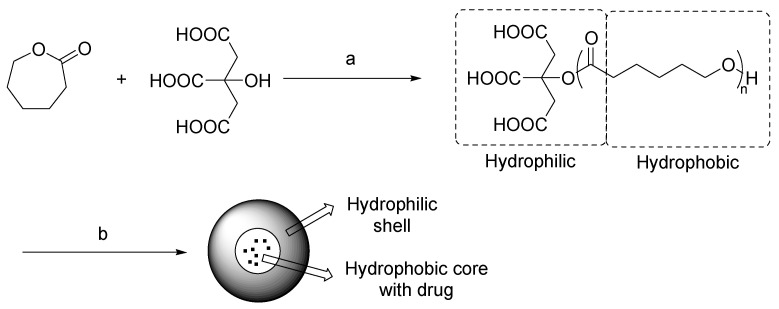
Condition reactions: (**a**) ChCl/citric acid (1:2), 120 °C, 24 h; (**b**) self-assembly, campthotecin.

In the last years, biocompatible materials for development of drug delivery systems have been synthesized by using NADES as sustainable reaction media [74]. An interesting study conducted by Pradeepkumar et al. described the preparation of a ε-caprolactone-citric acid micellar nanocarrier for controlled release of camptothecin [75]. In this procedure, the NADES acted both as a solvent and as a citric acid donor for the functionalization of caprolactone (Scheme 13). The structure of encapsulated micelles was analyzed by FT-IR and UV spectrometry, and biological studies were performed, showing a controlled release of camptothecin and time-dependent apoptosis. These results suggest that these micellar nanocarriers could represent a potent system for cancer therapy.

**Scheme 14:**
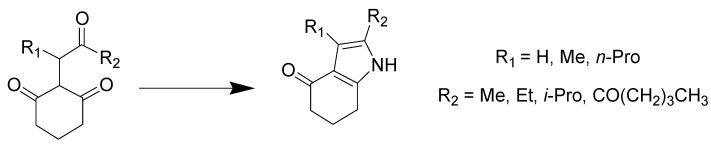
Condition reactions: ChCl/urea (1:2), 100 °C, 8 h.

Pyrrole is a five membered aromatic heterocycle present in complex macromolecules including porphyrins of heme and chlorophyll and in natural products. Pyrrole derivatives can serve as promising drug candidates with antimicrobial, antiviral, antimalarial, antitubercular, anti-inflammatory, and enzyme inhibiting activities [76]. Hu et al. reported the condensation reaction between tricarbonyl compounds and ammonia generated in situ from the eutectic mixture of ChCl/urea [77]. Triketones were completely transformed into pyrrole products with high yields at an optimum temperature of 100 °C, and the urea present in the DES was transferred to the final product of the reaction following its thermal decomposition (Scheme 14).

**Scheme 15:**
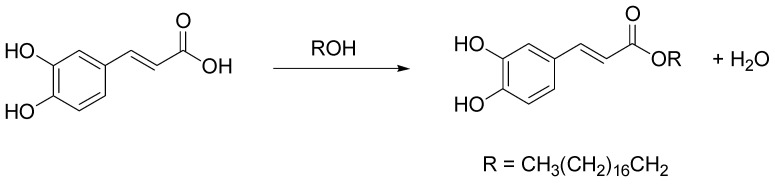
Optimized condition reactions: ChCl/caffeic acid, cation-exchange resin A-35 load 5%, 85 °C, 24 h.

Caffeic acid is a natural hydroxycinnamic acid phenolic derivative (3,4-dihydroxycinnamic acid), with antioxidant, anti-inflammatory, antimicrobial, and antineoplastic activities [78]. However, its unsatisfactory solubilities in both hydrophobic and hydrophilic systems limit its application in chemical industries. Considering that caffeoyl lipophilic alkyl esters show superior biological and pharmacological properties with respect to their phenolic acid counterparts, much research has focused on their preparation in chemical, biochemical, or enzymatic ways [79]. In this field, Wang et al. recently described a novel method for octodecyl caffeate production by using a NADES consisting of ChCl and caffeic acid as the caffeoyl donor, with cation-exchange resins as green catalysts and fatty alcohols (ROH) as caffeoyl acceptors (yield 91%) (Scheme 15) [80]. The proposed esterification mechanism involves the H^+^ released from the cation-exchange resin, that attacks the carboxyl carbon of caffeic acid to form a carbocation. Then, the OH of stearyl alcohol reacts with the carbocation, giving a tetrahedral intermediate, that releases H_2_O and H^+^ to form octodecyl caffeate.

## 4. Conclusions

In the last decades, scientific interest in the development of synthetic strategies involving green solvents has been strongly raised, sustained by the economic pressure to maximize process yields, and reduce production costs. This is particularly true in the pharmaceutical field, given the high value of the produced materials. In the present review, recent applications of ChCl-based DES as performing solvents in the synthesis of bioactive compounds were discussed. Most of the reported methodologies involve one-pot or multicomponent reactions; this kind of approach significantly reduced the amounts of solvents needed for intermediate purification. Our attention has focused on reactions in which ChCl-based DES are used as solvents, solvents/catalysts, or chemical donors. From our discussion, it emerged that the use of DES promoted the examined reactions from the point of view of yields, reaction times, and purification of the products. Therefore, ChCl-based DES can be suggested as valuable media in the synthesis of pharmaceuticals.

## Data Availability

No data are reported.
